# Preparation and Performance Study of Rapid Repair Epoxy Concrete for Bridge Deck Pavement

**DOI:** 10.3390/ma17112674

**Published:** 2024-06-01

**Authors:** Linhao Sun, Xinling Hao, Jilei He, Yingchun Cai, Pan Guo, Qingwen Ma

**Affiliations:** 1 Zhengzhou Public Utility Investment Development Group Co., Ltd., Zhengzhou 45003, China; 2 School of Water Conservancy and Transportation, Zhengzhou University, Zhengzhou 45001, China; 16638740739@163.com (X.H.);

**Keywords:** bridge deck pavement diseases, rapid repair of bridge deck pavement, epoxy concrete, thermal sensitivity testing, aging resistance

## Abstract

With the rapid development of bridge construction, the service life of bridges and traffic volume continue to increase, leading to the gradual appearance of diseases such as potholes and cracks in bridge deck pavements under repeated external loads. These issues severely impact the safety and service life of bridges. The repair of bridge deck potholes and cracks is crucial for ensuring the integrity and safety of bridge structures. Rapid repair materials designed for this purpose play a critical role in effectively and efficiently addressing these issues. In order to address the issues of pavement diseases, this study focuses on the rapid repair of epoxy concrete for bridge deck pavements and its performance is studied using experimental methods. Firstly, carbon black, rubber powder, and other materials were used to improve the elastic modulus and aging resistance of the epoxy concrete. Secondly, the addition of solid asphalt particles provided thermal sensitivity to the repair material. Finally, various properties of the rapid repair epoxy concrete for bridge deck pavements were tested through experiments including compressive strength testing, elastic modulus measurement, thermal sensitivity testing, and anti-UV aging testing. The experimental results show that adding carbon black and rubber powder reduces the elastic modulus of epoxy concrete by 25% compared to normal epoxy concrete, while increasing its aging resistance by 1.8%. The inclusion of solid asphalt particles provided thermal sensitivity to the repair material, contributing to better stress coordination between the repair material and the original pavement material under different temperature conditions. The epoxy concrete has early strength, toughness, and anti-aging properties, making it suitable for rapid repair of bridge deck pavement.

## 1. Introduction

With the rapid development of bridge construction, the proportion of bridges in highway construction is increasing, but with the development of the transportation industry, the increase in the service life of bridges and the traffic volume and the growing bridge deck pavement layer, diseases appear under the repeated action of the external load [1]. Pit is one of the most common diseases [2]. The appearance of potholes not only seriously affects the surface function and performance of the road surface, but also seriously affects the comfort and safety of driving. Moreover, with the growth of the service life of the bridge, the pit will further penetrate into the base, thus shortening the service life of the bridge. This ultimately affects the safety of the bridge, and even causes major accidents. Timely repair of the pit groove can not only restore the surface function of the road surface, ensure ride comfort and comfort when driving, but also restore the local strength and bearing capacity of the road surface in time. But because the bridge itself is in the road traffic network, it is impossible to completely close the traffic for a long time for bridge deck pavement repair. Therefore, we seek quick repair of the bridge deck pavement epoxy concrete [3] to ensure the normal traffic demand and maintain the performance and durability of the bridge deck pavement [4]. This has important implications.

Traditional bridge deck pit and groove repair technology. Most repair materials are sealed and classified according to the mixing temperature of the repair materials. The corresponding repair technology can be divided into thermal repair technology and cold repair technology [5,6]. Among them, the ordinary precast cold mixed repair technology generally adopts ordinary cold mixed asphalt concrete for sealing and filling, and the traditional hot repair technology adopts hot mixed asphalt concrete for sealing and filling. Compared with the hot repair technology, the cold repair technology does not need to heat the material, which is more in line with green traffic [7], which is also the development trend of the future bridge deck pavement rapid repair technology. Although the common precast cold mixed asphalt concrete repair technology has the advantages of construction at low temperature, local mixing, no heating, and energy saving, due to the rainy season and ultraviolet rays, the bridge deck pavement repair material life is not long and the construction cost is high. Although the traditional thermal repair technology can reach more than 1 year, it requires high construction conditions, heating asphalt and stone in advance, and will produce hot asphalt smell and smoke, which does not meet the requirements of green traffic.

The existing literature has explored pothole repair materials. Pang et al. [8] developed a new water-based epoxy concrete composite repair material (WECM) using synthesized water-based epoxy resin to meet construction requirements. Osama et al. [9] found that rubber cement-based composite materials as lightweight environmentally friendly concrete can efficiently repair bridge structures. Vishavkarma et al. [10] prepared a novel foam concrete for repairs using blast furnace slag and fly ash combinations, studying its mechanical properties. Jiusu et al. [11] validated the possibility of using SMA for repairing road potholes, highlighting the superior thermal conductivity of modified asphalt. Karimi et al. [12] discovered significant crack repair effects using polymer concrete repair materials as adhesives for all temperatures. Bruno et al. [13] explored the potential of utilizing hot recycling technology for producing patch asphalt for repairing potholes and practical cut locations. Luiz et al. [14] recommended verifying the bonding process before repairing high-strength concrete, ensuring compatibility with the concrete strength.

Many scholars at home and abroad have conducted research on crack repair materials. Karimi et al. [12] found that polymer concrete repair materials are significantly effective as adhesives for crack repair at all temperatures. Grzegorz et al. [15] studied the effects of silica fume, silica, and nano-silica on the morphology of the cement-based interface transition zone and crack size, modifying concrete with activated volcanic ash additives to uniformly reduce the crack size of concrete composite material structure. Ren et al. [16] used calcined coal gangue powder to partially replace cement and developed high-ductility, rapid-setting asphalt concrete overlay rapid repair materials. Si et al. [17] conducted three-point bending tests to study the crack propagation process in BDAP, finding that the presence of longitudinal cracks and decreasing temperature accelerate the propagation of reflective cracks. The crack resistance performance of ARHM-20 is better than ARHM-13, so ARHM-20 mix should be used in the lower layer of BDAP to limit the propagation of reflective cracks to the upper layer. Jiang et al. [18] found that when steel fibers and MgO are used together, the three-dimensional distribution of steel fibers further restricts the expansion of MgO, optimizes the concrete pore structure, improves concrete strength and durability, and solves issues such as shrinkage cracking on bridge decks.

Meanwhile, epoxy concrete, as a new type of repair material, has been the focus of research by scholars both domestically and internationally regarding its toughening techniques. Pang et al. [8] utilized self-synthesized water-based epoxy resin (WEP) mixed with WEP fibers and embedded cement particles to provide fiber protection for water-based epoxy concrete composite repair materials, increasing their toughness by 300~600%. Shao et al. [19] found that epoxy concrete with added rubber particles can serve as repair material, enhancing the toughness of the epoxy concrete. Byung et al. [20] discovered that incorporating nano-clay and carbon black into epoxy resin at room temperature can enhance the toughening effect of the epoxy resin system. Flores et al. [21] modified epoxy resin and anhydride curing agents using hyperbranched polyesters to improve the toughness of the epoxy resin system.

Many scholars have conducted extensive research on the aging resistance of repair materials. Zhou et al. [22] added surfactant warm mix additives to common MgAl-layered double hydroxide materials, enhancing the anti-UV aging performance of road materials. Cho et al. [23] improved the aging resistance of concrete by utilizing fly ash volcanic ash reaction filling effect internally. Qu et al. [24]. found that adding nano-SiO_2_ and nano-TiO_2_ reduced the damage of UV aging conditions to water-based coatings, with nano-TiO_2_ showing better improvement effects than nano-SiO_2_. Xu et al. [25] studied the aging resistance of polyether-type polyurethane concrete (PPC), showing that PPC has good aging resistance, and its road performance after aging is better than SBS-modified asphalt mixtures that are not aged. Yu et al. [26] confirmed that highly SBS-modified asphalt has better aging resistance compared to slightly SBS-modified asphalt. Babu et al. [27] enhanced the aging resistance of VG30 asphalt binder by adding Sasobit (warm mix additive) and hydrated lime.

To ensure the stress coordination between repair materials and original pavement materials at different temperatures, research has been conducted domestically and internationally on the thermal sensitivity of concrete. Shekhovtsova et al. [28] proposed a method to calculate the boundary layer thickness of the “asphalt-dispersed phase” binary system, concluding that the strength of composite materials increases with the increase in the structural asphalt layer thickness. They introduced the generalized factor m index reflecting the interfacial area impact and the reinforcement factor n index reflecting the impact of physicochemical effects at the interface to evaluate the sensitive performance impact of composite material structures, finding that silica surface area can improve the thermal sensitivity of asphalt concrete. Anmar et al. [29] developed a new rapid maintenance environmentally friendly cold asphalt concrete binder mixture (CACB) by using particle size distribution similar to traditional hot mix asphalt concrete mixture and introducing binary blended cementitious filler (BBCF), showing that the addition of alkali-activated binary blended cementitious filler significantly improves concrete thermal sensitivity. Edoardo et al. [30] confirmed that epoxy asphalt concrete (EAC) has superior thermal sensitivity compared to conventional hot mix asphalt concrete.

To sum up, there are still some deficiencies in the existing bridge deck pavement repair materials: (1) the research on rapid concrete repair materials currently focuses on the repair of cement concrete. Cement concrete repair material can not meet the requirements of asphalt bridge deck pavement for the consistent color. (2) The existing asphalt concrete repair materials cannot meet the requirements of rapid repair of asphalt bridge deck pavement. (3) Ordinary epoxy concrete does not have aging resistance. It will lead to the problem of poor durability in the engineering application of bridge deck pavement repair. (4) Epoxy concrete strength, high stiffness. It is easy for it to look bad with the surrounding old shop cooperative deformation and secondary damage and other problems. Based on this, an epoxy concrete that can meet the requirements of rapid repair of asphalt concrete bridge deck pavement is urgently needed [31]. In order to maintain the service performance and durability of asphalt concrete bridge deck pavement.

In view of the above problems, this paper studies the rapid repair of epoxy concrete on bridge deck, and uses black aggregate to improve the color of epoxy concrete and make it with asphalt concrete [32], close in color. Rubber powder is added to reduce the elastic modulus of epoxy concrete. Carbon black is added to improve the anti-aging performance of epoxy concrete. Solid asphalt particles are added to the heat-sensitive repair materials to ensure the force coordination with the original pavement materials under different temperature conditions, so as to meet the technical requirements of rapid repair of bridge deck pavement.

## 2. Preparation of Epoxy Resin Concrete Specimens

### 2.1. Raw Materials

#### 2.1.1. Epoxy Resin, Thinner and Curing Agent

Common epoxy resin are roughly divided into the following categories: bisphenol A type epoxy resin, bisphenol F epoxy resin, polyphenol glycidyl ether epoxy resin, aliphatic glycidide epoxy resin, glycidide epoxy resin, epoxide olefin compounds and heterocyclic and mixed epoxy resin. Among them, bisphenol type A epoxy resin is used as a construction adhesive due to its low cost, good process performance, high yield, excellent mechanical properties and good durability [8]. Since E51 bisphenol A epoxy resin has the highest epoxy and the smallest epoxy equivalent, E51 epoxy resin was used for this test. The epoxy resin used in this experiment is from Kunshan Jiulimei Electronic Materials Co., Ltd. (Kunshan, China). Table 1 shows the technical indexes of E51 type epoxy resin.

Epoxy resin diluents are mainly divided into active thinner and inactive diluents. Among them, the active diluent of No [33] can directly participate in the curing reaction of epoxy resin and become a part of the crosslinking network structure of epoxy resin curing material, with almost no effect on the performance of the curing products. Moreover, C12–14 fat glycidide ether has the characteristics of shallow color, acceptable smell, low toxicity, good fluidity and excellent flexibility. Therefore, C12–14 fat glycidyl ether was used as an epoxy resin diluent in this test. C12-14 fatty alkyl epoxy ether is provided by Zhengzhou Penghui Chemical Products Co., Ltd. (Zhengzhou, China). Table 2 shows the technical index of C12–14 fat glycidyl ether thinner.

Considering that the temperature change will affect the setting time and strength of the material, and then affect the stability and durability of the pavement layer, in order to solve the influence of the temperature change on the pavement material, this test uses different kinds of curing agents for compound to enhance the performance of the pavement material. Polyester-type low-temperature curing agent [34] can cure by a low-temperature and epoxy resin reaction, and amine normal temperature curing agent can quickly cure at room temperature; therefore, the polyester-type low-temperature curing agent and amine normal temperature curing agent have the advantages of low-temperature curing and room-temperature curing, ensuring the stability and durability of bridge deck pavement materials at different temperatures. The polyester-type low-temperature curing agent is provided by Guangzhou Gelingshi New Materials Co., Ltd. (Guangzhou, China), and the amine normal temperature curing agent is provided by Kunshan Jiulimei Electronic Materials Co., Ltd. (Kunshan, China).

#### 2.1.2. Aggregate

**Figure 1:**
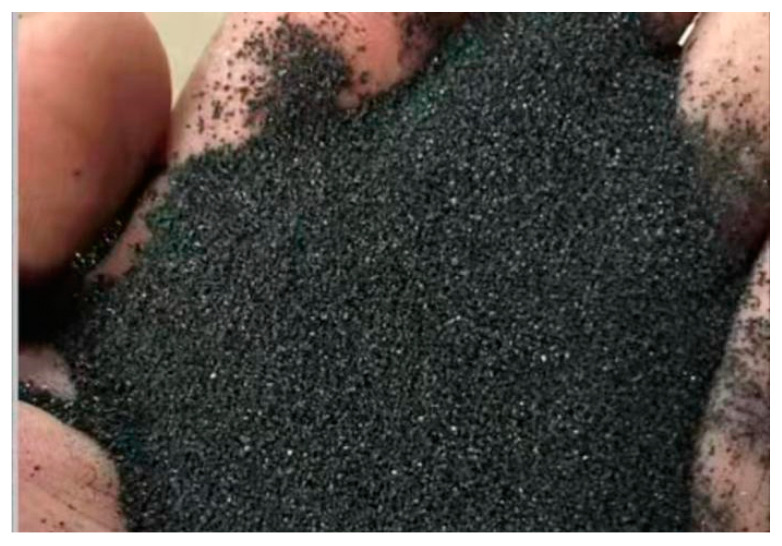
Black emery.

In order to meet the basic requirements for asphalt bridge deck pavement repair, black aggregates were used in this experiment to ensure that the color of the epoxy concrete obtained is similar to that of asphalt concrete. Considering the low cost, good wear resistance, strong impact resistance, excellent compressive strength, and color options of silicon carbide, black silicon carbide was selected as the main aggregate [35], as shown in Figure 1. Additionally, carbon black [36] was added to ensure the anti-aging performance of the bridge deck pavement. Rubber powder was mixed in to improve the material’s toughness and reduce the elastic modulus. Solid asphalt particles were added to make the repair material thermosensitive and to better ensure stress coordination between the repair material and the original pavement material under different temperature conditions.

The material ratio was determined as follows: mass ratio of epoxy resin to curing agent to diluent = 1.0:1.0:0.1; mass ratio of aggregate to gel system = 3.0:1.0. The black silicon carbide used consisted of three particle sizes: 8–10 mesh accounted for 10–30%, 20–40 mesh accounted for 10–30%, and 2–4 mesh accounted for 10–30%. Carbon black with a fineness of 100–120 mesh is used in a proportion of 0–2 parts, while rubber powder with a fineness of 100–120 mesh is used in a proportion of 0.5–6 parts. As shown in Table 3, the asphalt particles used were 80–100 mesh with a ratio of 5–15%.

Black silicon carbide is provided by Zhengzhou Jindou Clean Water Materials Co. (Zhengzhou, China), Ltd., carbon black is provided by Jinan Zhongbei Chemical Co., Ltd. (Jinan, China), rubber powder is provided by Zibo Jiubao Powder Factory (Zibo, China), and asphalt particles are provided by Zhengzhou Chunyuan Chemical Co., Ltd. (Zhengzhou, China).

### 2.2. Instruments and Equipment

**Figure 2:**
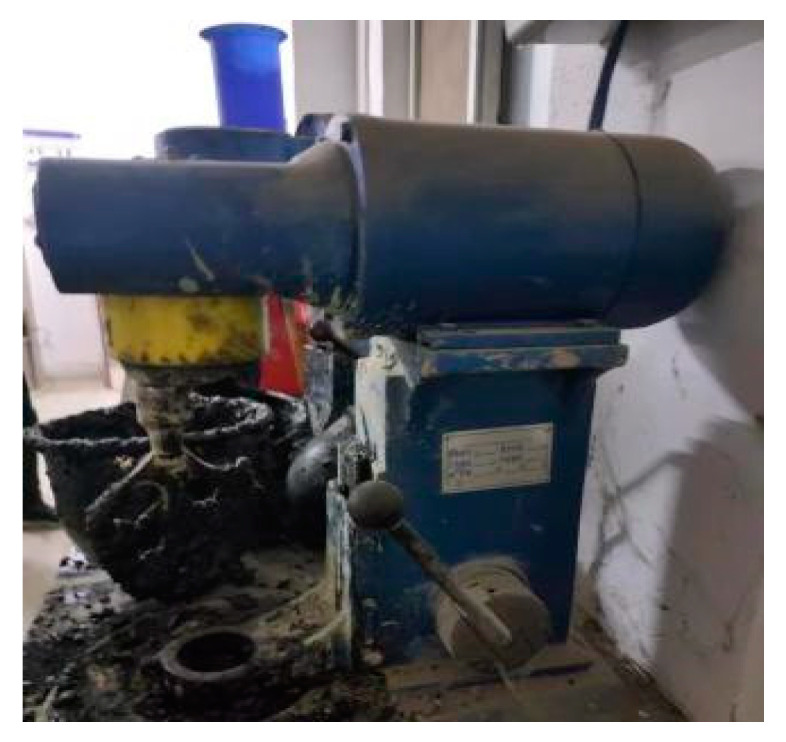
Cement mixer.

**Figure 3:**
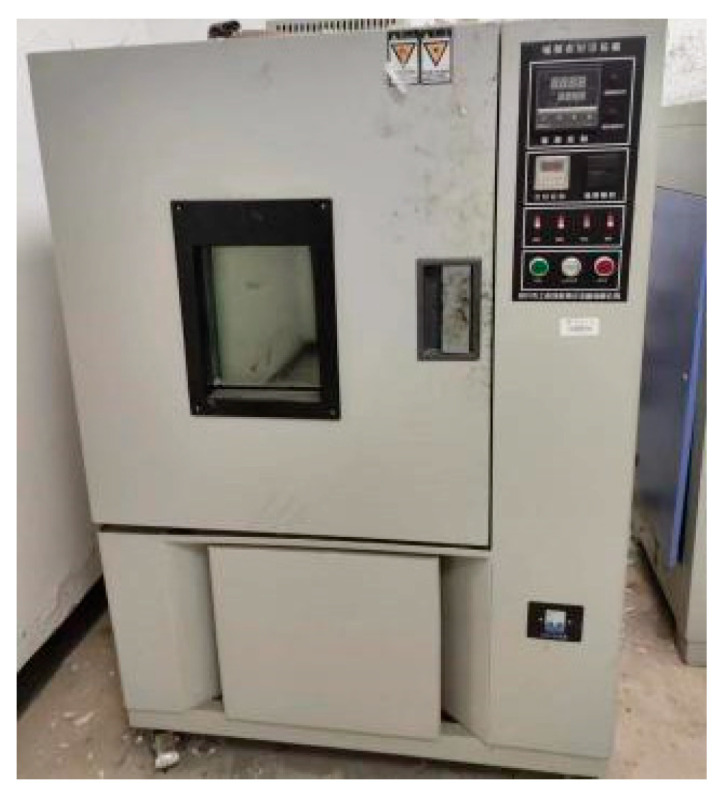
Irradiation aging test box.

**Figure 4:**
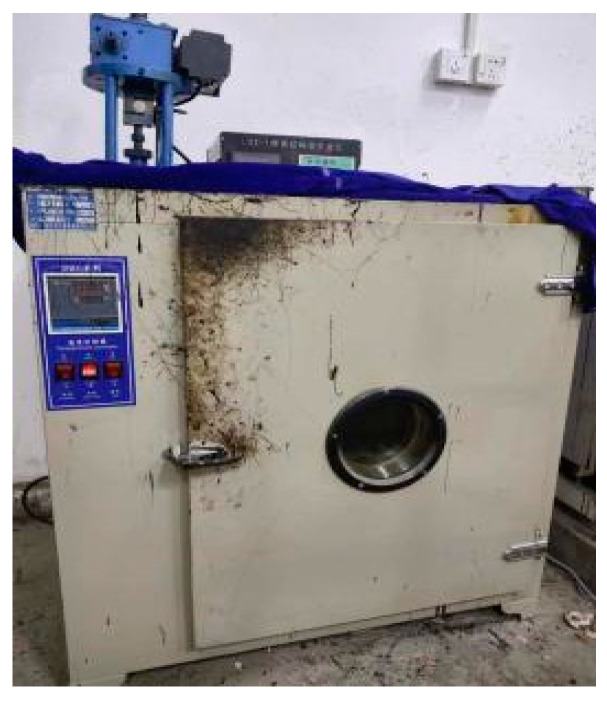
Electric heating constant temperature drying box.

**Figure 5:**
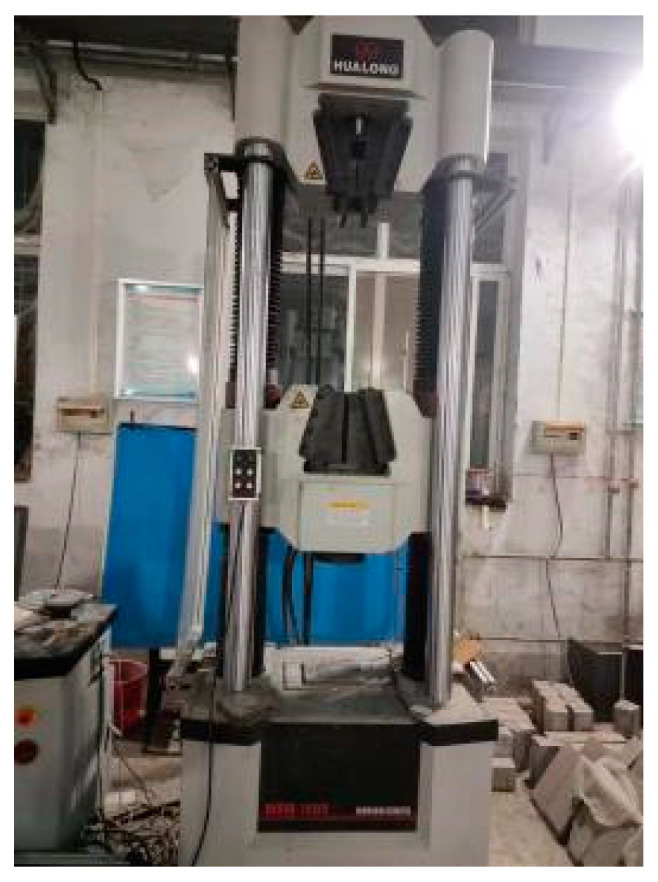
Universal tester.

The main test equipment used in this test includes a cement mixer, a irradiation aging test box, an electric thermostatic drying box and a microcomputer control o-hydraulic servo universal test machine. The main technical indicators are shown in Table 4, and the specific styles of all kinds of test equipment are shown in Figure 2, Figure 3, Figure 4 and Figure 5.

### 2.3. Sample Preparation

During the preparation of epoxy concrete, the weights of each component of the concrete material, including resin, curing agent, diluent, and aggregate, were calculated and measured according to the material ratio. Accurately measuring the weight of each component ensures the accuracy and consistency of the epoxy concrete mixture. Next, the epoxy resin, curing agent, and diluent were added to the mixing bowl of the cement mortar mixer and uniformly mixed to ensure thorough blending and formation of a uniform mixture. Finally, the aggregate was gradually added to the mixing bowl for uniform stirring to ensure thorough integration with the resin mixture and formation of a uniform concrete mass.

**Figure 6:**
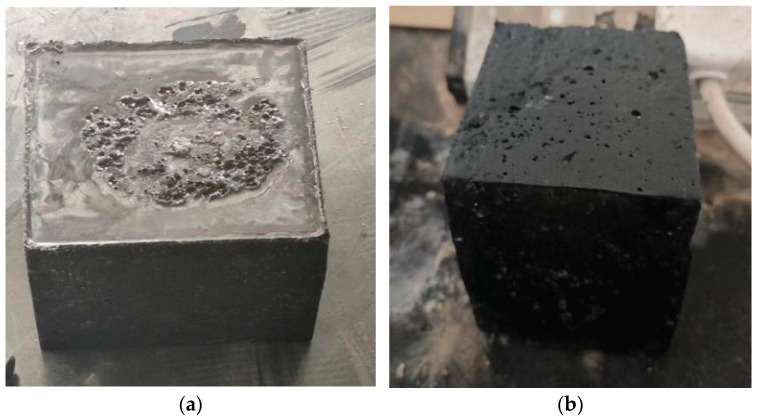
Epoxy concrete cube. (**a**) Type I epoxy concrete cube test piece measuring 100 × 100 × 100 mm. (**b**) Type II epoxy concrete cube test piece measuring 100 × 100 × 100 mm.

**Figure 7:**
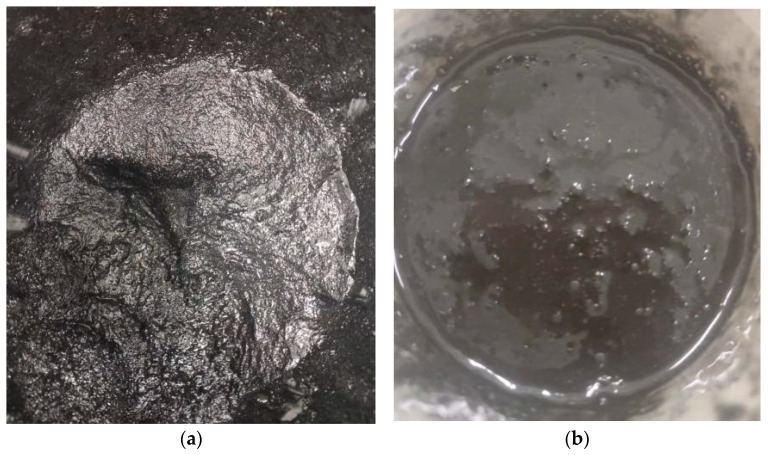
Epoxy concrete mixing process. (**a**) Epoxy concrete during the mixing process. (**b**) Epoxy concrete after mixing.

In this test, the materials were mixed evenly to the mix, and then the liquid part and aggregate were mixed in the mix for 3 min until evenly mixed, and put into the cast iron molds of 100 mm × 100 mm × 100 mm and 100 mm × 100 mm × 100 mm × 300 mm, flattened on the vibrator and solidified under the standard conditions. Table 5 is the working condition table of epoxy concrete test, and some specimens are shown in Figure 6a,b. The epoxy concrete during the mixing process is shown in Figure 7a,b including the specimen and the early compressive strength [37].

### 2.4. Test Method

#### 2.4.1. Compressive Strength of Cubic Specimens

The determination method of compressive strength of cubic specimens test refers to GB/T 50081-2019 “Test methods for mechanical properties of concrete” [38], with specific steps as follows.

**Figure 8:**
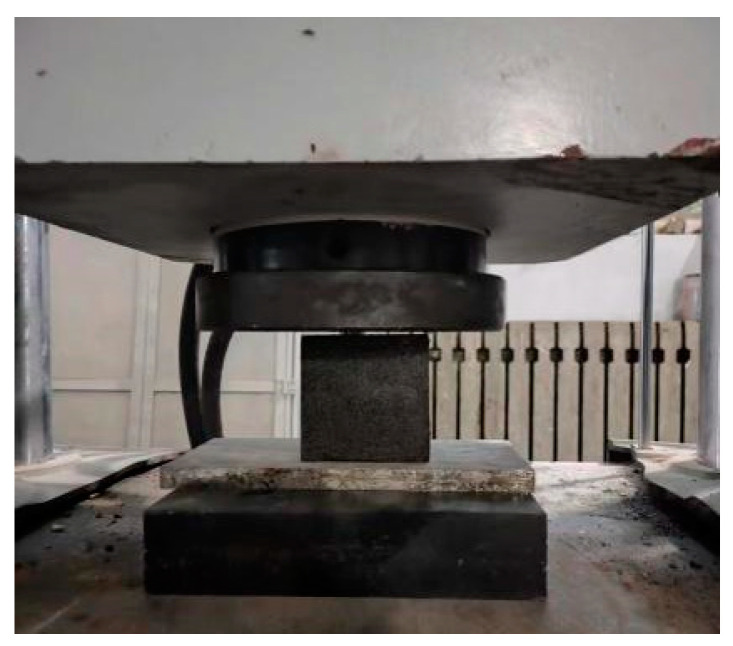
Cube compressive strength test.

(1) When the specimen reaches the test age, it should be taken out from the curing location, and its dimensions and shape should be checked. The dimensional tolerance should not exceed 1 mm. The specimen should be tested as soon as possible after being taken out.
(2) Before placing the specimen in the testing machine, wipe the surface of the specimen clean with the upper and lower compression plates.
(3) The side surface of the specimen formed during molding should be the compression surface. Place the specimen on the lower compression plate or cushion plate of the testing machine, and align the center of the specimen with the center of the lower compression plate of the testing machine, as shown in Figure 8.
(4) Start the testing machine, ensuring that the surface of the specimen is in uniform contact with the upper and lower compression plates or steel cushion plates.
(5) During the experiment, load should be continuously and uniformly applied, with a loading speed of 0.3–0.5 MPa/s. When the compressive strength of the cubic specimen is less than 30 MPa, the loading speed should be 0.3–0.5 MPa/s. When the compressive strength of the cubic specimen is 30–60 MPa, the loading speed should be 0.5–0.8 MPa/s. When the compressive strength of the cubic specimen is not less than 60 MPa, the loading speed should be 0.8–1.0 MPa/s. The faster the test speed, the lower the material’s strength results may become because rapid application of pressure can lead to faster deformation and damage within the material. On the contrary, slower test speeds may result in higher strength results because the material has more time during the test process to adapt to the pressure and demonstrate its true strength. Therefore, it is important to choose the appropriate test speed based on specific circumstances when conducting pressure tests to ensure reliable results. This is because under high-speed loading conditions, the internal microstructure of the test specimen may not be able to adapt to the external pressure applied in a timely manner, leading to instantaneous changes or damage in internal microlevel or grain structures, thereby affecting the determination of compressive strength results. Additionally, the deformation rate effect in the test specimen may be induced by high-speed loading, where deformation and stress concentration occurring in a short period of time may lead to faster crack propagation and material damage, resulting in lower measured compressive strength values. Furthermore, the dynamic response of the specimen to the test speed can also have an impact, as rapid loading may induce dynamic effects within the specimen, such as strain rate effects and impact loading effects, which may cause non-linear changes in the stress–strain response of the specimen, thereby affecting the accuracy of strength test results [39].
(6) When manually controlling the loading speed of the pressure machine, when the specimen is close to the point of drastic deformation at the beginning of failure, stop adjusting the throttle of the testing machine until failure occurs, and record the failure load. The compressive strength of concrete cubic specimens is calculated according to Formula (1) [38].

(1) $$f_{cu}=\frac{F_{cu}}{A_{1}}$$

where

$f_{cu}$—compressive strength, MPa.
$F_{cu}$—failure load, N.
$A_{l}$—area under compression, mm^2^.

#### 2.4.2. Compressive Strength of Cubic Specimens

The method for determining the static compression elastic modulus test complies with GB/T 50081-2019 “Test methods for physical properties of concrete”. The specific steps are as follows.

(1) When the specimen reaches the test age, it should be removed from the curing location, inspecting its dimensions and shape. The dimensional tolerance should not exceed 1 mm, and the test should be conducted as soon as possible after the specimen is removed.
(2) When determining the concrete elastic modulus, the microdeformation measurement instrument should be installed on the midline of the specimen on both sides and symmetrically at the two ends of the specimen. When using dial indicators or displacement sensors, the dial indicators or displacement sensors should be fixed on the deformation measurement frame, and the measuring distance of the specimen should be 150 mm, positioned by the locating rod. The deformation measurement frame should be secured with fastening screws. When using strain gauges to measure deformation, the gauge length of the strain gauges should be 150 mm. After removing the specimen from the curing chamber, any surface defects in the area where the strain gauges are applied should be treated using a hairdryer to dry the surface of the specimen and attaching the strain gauges with 502 glue in the middle of the specimen on both sides.
(3) Before placing the specimen on the testing machine, the surface of the specimen should be wiped clean with the upper and lower pressure plates.
(4) The specimen should be placed upright on the lower pressure plate or steel cushion plate of the testing machine, aligning the center of the specimen with the center of the lower pressure plate.
(5) The test machine should be started ensuring that the surface of the specimen is evenly in contact with the upper and lower pressure plates or steel cushion plates.
(6) The initial load value should be increased to a basic stress value of 0.5 MPa, maintaining the load for 60 s and recording the deformation readings of each measurement point within the next 30 s. The load should then be continuously and uniformly increased to one-third of the compressive strength of the specimen’s axial stress, maintaining the load for 60 s and recording the deformation readings of each measurement point within the next 30 s. Loading should be continuously and evenly applied during the experiment, with a loading speed of 0.3–1.0 MPa/s. When the cube compressive strength is less than 30 MPa, the loading speed should be 0.3–0.5 MPa/s. When the compressive strength is 30–60 MPa, the loading speed should be 0.5–0.8 MPa/s, and when the compressive strength is not less than 60 MPa, the loading speed should be 0.8–1.0 MPa/s.
(7) If the difference between the deformation values on the left and right sides ratio and their average value is greater than 20%, the specimen should be realigned and the provisions of step (6) repeated. If it cannot be reduced to less than 20%, the test is invalid.
(8) After confirming that the specimen is re-aligned in accordance with the 8th paragraph of this article, the load should be unloaded to the basic stress of 0.5 MPa at the same speed, maintaining it for 60 s. The same loading and unloading speed, and maintaining the load for 60 s, should be repeated at least twice for repeated preloading. After the final preloading is complete, the load should be maintained at 0.5 MPa for 60 s and the deformation readings recorded for each measurement point within the next 30 s. Then, the load should be applied at the same loading speed and maintained for 60 s, recording the deformation readings for each measurement point within the next 30 s.
(9) Remove the deformation measurement instrument and apply the same speed loading until failure, recording the failure load.
(10) Calculate the concrete static compression elastic modulus according to Formulas (2) and (3) [38].

(2) $$E_{C}=\frac{F_{a}-F_{0}}{A_{3}}\times \frac{L}{\Delta n}$$

(3) $$\Delta n=\varepsilon_{a}-\varepsilon_{0}$$

where

$E_{c}$—elastic modulus, MPa.
$F_{a}$—load when the stress is 1/3$f_{cp}$, N.
$F_{0}$—load at stress is 0.5 MPa, N.
$A_{3}$—test piece pressure area, mm^2^.
L—measure the distance, 150 mm.
Δn—the average value of the deformation of the specimen from the last load to, mm.
$\varepsilon_{a}$—the average value of the deformation on both sides of the specimen, mm.
$\varepsilon_{0}$—the average value of the deformation on both sides of the specimen, mm.

## 3. Results and Analysis

### 3.1. Compressive Strength of the Cube

Testing the compressive strength of specimens with different curing times, we analyze the trend of epoxy concrete compressive strength over time. The specific experimental results are shown in Table 6. Both types of epoxy concrete show a gradual increase in compressive strength with increasing age, attributed to the hydration reaction of the epoxy resin and hardener in the epoxy concrete, forming a network structure of crosslinks. With the increase in age, this three-dimensional crosslinking structure gradually improves, enhancing the bonding force between molecules and thus increasing the overall strength of the concrete. Moreover, as the age progresses, the molecules in the epoxy concrete will gradually align into a more orderly structure, improving the mechanical properties of the material. The arrangement and bonding between molecules make the material denser and more organized, thereby enhancing the concrete strength. During the solidification process, epoxy concrete undergoes crosslinking reactions to form a fixed three-dimensional structure. With the increase in age, this crosslinking structure continues to strengthen, and the reduction in microscopic defects within the material leads to a gradual increase in the compressive strength of epoxy concrete [40]. Specifically, the compressive strength of Type I epoxy concrete cubes increases with age, rising from 10 MPa to 33 MPa, while the strength of Type II epoxy concrete increases with age from 12 MPa to 39 MPa.

Standard deviation analysis is a statistical method used to measure the dispersion or volatility of data. By calculating the standard deviation from the mean, one can observe the average deviation between the observed values in a data set and the mean. Standard deviation analysis of the compressive strength of two types of epoxy concrete reveals that the standard deviation of compressive strength for the first type ranges from 1.00 to 3.61 MPa, while the second type has a consistent standard deviation of 2.65 MPa. This indicates that the compressive strength of both types of epoxy concrete is relatively stable.

**Figure 9:**
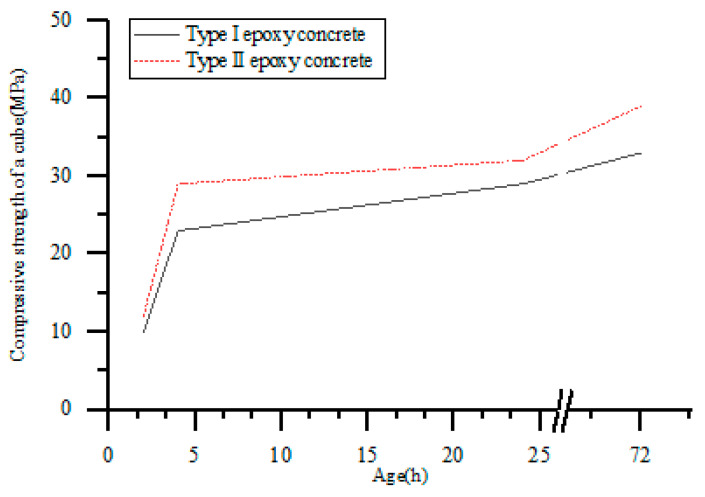
Relation between cube compressive strength and age of epoxy resin concrete.

As shown in Figure 9, the compressive strength of epoxy concrete with carbon black and powder is higher than that of epoxy concrete with no carbon black and powder. This is because the large rubber molecules can slide on the carbon black, and the surface activity of the carbon black particles is inconsistent. The rubber chains adsorbed on the surface of the carbon black can gain different binding energies. In contrast, epoxy concrete has a nearly perfect dense structure, where rubber and carbon black particles are encapsulated by epoxy resin and adhere to the surrounding epoxy matrix. The rubber and carbon black particles serve as stress centers to induce crack formation and shear bands, thereby absorbing a large amount of deformation energy and enhancing the strength of epoxy concrete [19]. The carbon black and powder enhance the structural stability of epoxy concrete to a certain extent, thus improving its compressive strength [41]. With the growth of the age period. The increasing trend of the cubic compressive strength slows down. Probably because the chemical bonds of the epoxy concrete have been formed and reinforced to a certain extent. With the increase in age, the increase in compressive strength will decrease. Two types of epoxy resin concrete have been cured at 2 h age, compressive strength up to 10 MPa. The compressive strength of 3 d age has reached above 30 MPa. Specifically, the compressive strength of Type II epoxy concrete compared to Type I epoxy concrete increases from 10 MPa to 12 MPa at 2 h, from 23 MPa to 29 MPa at 4 h, from 29 MPa to 32 MPa at 1 day, and from 33 MPa to 39 MPa at 3 days. This indicates that the epoxy concrete solidifies with a fast rate, high compressive strength, which can be used as a quick repair material for bridge deck pavement. And the epoxy resin concrete with carbon black and rubber powder can meet the requirements of rapid repair for the bridge deck.

### 3.2. Elastic Modulus

**Figure 10:**
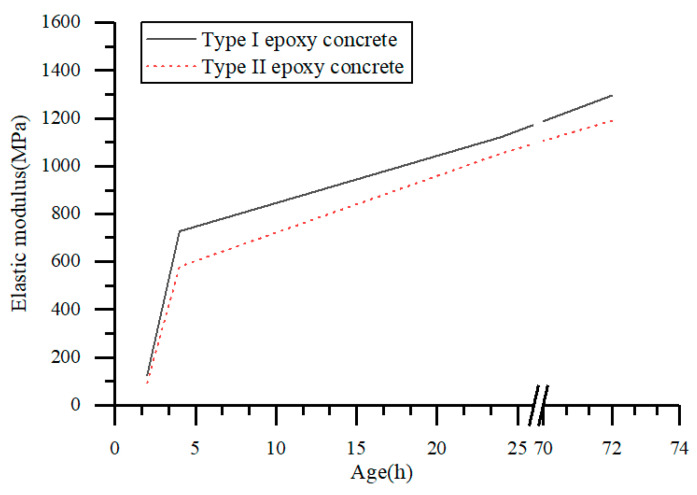
Relationship between elastic modulus and age of epoxy resin concrete.

The results of the static compressive elastic modulus test of epoxy concrete are shown in Table 7 and Figure 10. Both types of epoxy concrete exhibited a gradual increase in static compressive elastic modulus with age. Due to the crosslinking reaction of epoxy resin molecular chains in epoxy concrete to form a three-dimensional network structure, as the age increases, these crosslinking structures will continue to be refined and strengthened, leading to an increase in the material’s rigidity. More crosslinking points can increase the interaction forces between molecular chains, making the overall material more solid and rigid. With the increase in age, the molecular arrangement of epoxy concrete becomes more ordered, and partial crystallization processes occur. This ordered arrangement and crystallization enhance the material’s strength and rigidity, thereby increasing the elastic modulus. As the age increases, the microscopic defects such as pores and cracks in epoxy concrete gradually decrease, reducing the stress concentration within the material, decreasing energy dissipation, and consequently increasing the stiffness and elastic modulus of the material [40]. Specifically, the elastic modulus of Type I epoxy concrete increases with age from 125.43 MPa to 1294.32 MPa, while the elastic modulus of Type II epoxy concrete increases from 94.12 MPa to 1190.41 MPa with age.

An analysis of the standard deviations of the elastic modulus of two types of epoxy concrete reveals that the standard deviation of the elastic modulus of the first type of epoxy concrete ranges from 0.28 to 3.09 MPa, while the standard deviation of the elastic modulus of the second type of epoxy concrete falls between 1.56 and 3.45 MPa. Both types of epoxy concrete demonstrate relatively stable elastic modulus values. Epoxy concrete with added carbon black and rubber powder had lower elastic moduli compared to epoxy concrete without these additives. Carbon black and rubber powder can alter the microstructure of concrete, manifested by rubber particles being enclosed by epoxy resin with a dense structure and adhering to the surrounding epoxy matrix. Rubber particles serve as stress centers that induce crack and shear band formation, absorbing a significant amount of deformation energy and enhancing the fracture toughness of epoxy concrete. The increase in crack branching and interlocking around carbon black particles also enhances the fracture toughness of epoxy concrete. Rubber macromolecules adsorb onto different adsorption sites on the surface of carbon black, and their combined action makes epoxy concrete more flexible, thereby reducing its elastic modulus [20]. This reduction in elastic modulus is crucial for bridge deck repair work. Specifically, compared to Type I epoxy concrete, the elastic modulus of Type II epoxy concrete decreases from 125.43 MPa to 94.12 MPa at 2 h, from 727.43 MPa to 579.42 MPa at 4 h, from 1121.32 MPa to 1053.10 MPa at 1 day, and from 1294.32 MPa to 1190.41 MPa at 3 days. During the bridge deck repair process, it is essential for the new repair materials to effectively bond with the existing pavement materials to ensure overall pavement performance. If the elastic modulus of the new repair material is too high, it may lead to stress incompatibility with the existing material, affecting the performance of the bridge deck. Therefore, reducing the elastic modulus of epoxy concrete by adding carbon black and rubber powder is beneficial to ensure the coordination of stress between the rapid repair material for bridge deck paving and the original pavement material, thus maintaining the performance of the bridge deck. This approach not only improves the effectiveness of bridge deck repair but also extends the service life of the bridge deck, providing significant practical value.

### 3.3. Anti-UV Aging Properties

In order to test the impact of ultraviolet (UV) radiation on the compressive strength and the elastic modulus of epoxy concrete, comparative UV aging tests were conducted on two types of epoxy concrete under equal conditions. The specific operational steps were as follows: the fabricated epoxy concrete specimens were cured at room temperature for 24 h and then demolded. The concrete specimens were placed in a UV aging chamber and aged for two days under PV 42.2 °C and SV 45.0 °C conditions to simulate the exposure of epoxy concrete to UV radiation in actual use in order to investigate the effects of UV radiation on its performance. The specimens were then subjected to compressive strength and elastic modulus tests.

**Figure 11:**
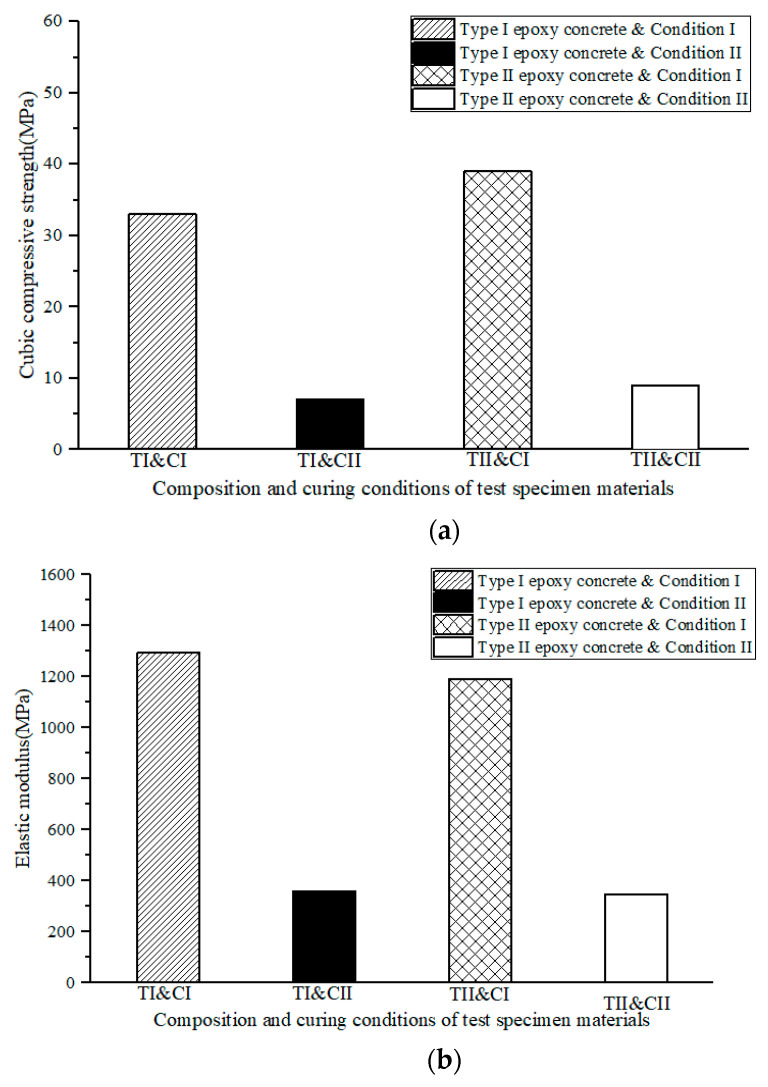
Compressive strength and elastic modulus of static pressure under different conditions. (**a**) Compressive strength. (**b**) Elastic modulus. Note: T I, T II, C I and C II are shown in Table 10.

**Figure 12:**
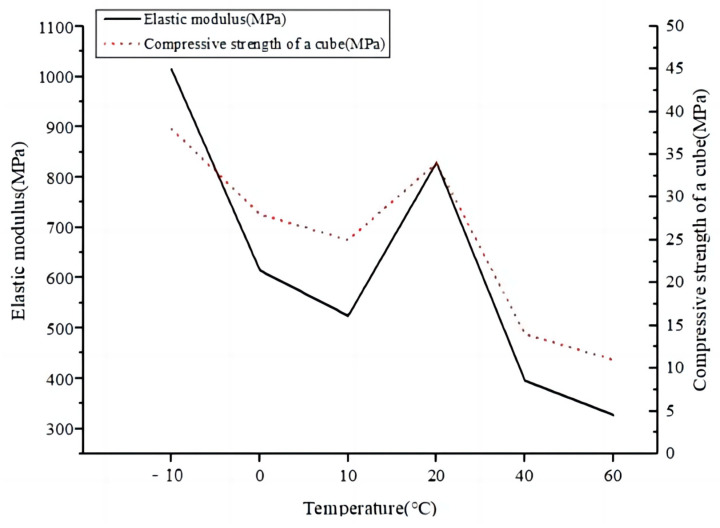
Compressive strength and static compression elastic modulus of the third type of epoxy concrete cube at different temperatures.

The compressive strength and the elastic modulus of cube specimens of the two types of epoxy concrete after exposure to UV radiation were determined. The test results are presented in Table 8 and Table 9, and Figure 11 and Figure 12. The acronyms in Figure 11 are specified as shown in Table 10. Under UV conditions, the compressive strength of epoxy concrete with added carbon black and rubber powder decreased by 76.9%. This is because rubber carbon molecules have a conjugated structure, where carbon atoms form a structure similar to an aromatic ring through π electron conjugation. This conjugated structure can absorb ultraviolet light within a certain wavelength range, and the π electrons in carbon black molecules can undergo electronic transitions under ultraviolet irradiation, absorbing ultraviolet energy. Additionally, carbon black is filled in epoxy concrete, forming a barrier that can reduce the erosion of ultraviolet light on the concrete material, thereby minimizing damage from ultraviolet light. This absorption capacity helps protect materials such as rubber from the aging effects of ultraviolet light, extending their lifespan [42]. Analyzing the data in the table, it can be observed that under ultraviolet conditions, the compressive strength of epoxy concrete with added carbon black and rubber powder decreased by 76.9%, while the compressive strength of epoxy concrete without these additives decreased by 78.8% under the same conditions. Specifically, the cube compressive strength of Type I epoxy concrete under standard conditions is 33 MPa, while under UV aging conditions, it decreases to 7 MPa. The cube compressive strength of Type II epoxy concrete under standard conditions is 39 MPa, but under UV aging conditions, it decreases to 9 MPa. Conducting a standard deviation analysis on the compressive strength and the elastic modulus of the two types of epoxy concrete under different conditions, the values are between 1.00 and 4.00 MPa, indicating that both types of epoxy concrete exhibit stability under different conditions. While the compressive strength of epoxy concrete without these additives decreased by 78.8% under UV conditions. Epoxy concrete with added carbon black and rubber powder exhibited higher strength than epoxy concrete without these additives, while the elastic modulus was lower. This indicates that epoxy concrete with added carbon black and rubber powder has better compressive performance and toughness under UV conditions, which is advantageous for ensuring the UV aging resistance of rapid repair materials for bridge deck paving. This can improve the service life of bridge deck paving to a certain extent, meeting the needs of rapid bridge deck repair and providing important references for engineering practice.

### 3.4. Thermal Sensitivity

The thermal sensitivity of epoxy concrete affects its performance under different temperature conditions. In order to ensure the stress coordination between repair materials and original pavement materials under different temperature conditions and optimize the performance of epoxy concrete, this experiment chose to improve its thermal sensitivity by adding solid asphalt particles.

The fabricated epoxy concrete specimens were cured at room temperature for 24 h, demolded, and then cured at room temperature for 3 days to ensure the strength and stability of the specimens and the accuracy of the experimental results. The concrete specimens were placed under conditions of −10 °C, 0 °C, 10 °C, 20 °C, 40 °C, and 60 °C to simulate the different temperature environments that epoxy concrete may encounter in practical applications, and their performance was tested.

By conducting compressive strength and elastic modulus tests on the epoxy concrete specimens at different temperatures, the impact of asphalt particles on the thermal sensitivity of epoxy concrete can be understood. The test results are presented in Table 11 and Table 12 and Figure 12. The mechanical performance of the third type of epoxy concrete with added solid asphalt particles varied at different temperatures. Specifically, when the temperature ranged from −10 °C to room temperature, the 3-day cube compressive strength was similar to that at room temperature. However, when the temperature exceeded 40 °C, the cube compressive strength decreased. This is because the micromolecules of asphalt particles themselves are complex organic compounds composed of carbon, hydrogen, oxygen, and other atoms, with a polycyclic aromatic structure. This molecular structure allows the micromolecules of asphalt to undergo structural changes and movement under the action of heat, exhibiting thermal sensitivity. The asphalt micromolecules also exhibit certain intermolecular forces, which weaken at high temperatures, making the asphalt micromolecules more prone to flow. Additionally, the softening point of asphalt micromolecules is usually relatively low, so when heated, the asphalt micromolecules reach their softening point and exhibit thermal sensitivity. When asphalt molecules fill the tiny pores in epoxy concrete, it increases the density and viscosity of the epoxy concrete, thereby affecting the thermal conductivity of the concrete. At the same time, interactions occur between the asphalt molecules and the cement matrix in the concrete, forming a stable interface that effectively reduces moisture infiltration and damage in the concrete, leading to improved heat resistance and durability of the concrete. Furthermore, asphalt molecules possess a certain degree of heat stability, which stabilizes the thermal conductivity of concrete, making the concrete exhibit better heat resistance and durability under high-temperature conditions [30]. Specifically, the cube compressive strength of Type III epoxy concrete is 38 MPa at −10 °C, 28 MPa at 0 °C, 25 MPa at 10 °C, 34 MPa at 20 °C, 14 MPa at 40 °C, and 11 MPa at 60 °C. Standard deviation analysis of the compressive strength and the elastic modulus of Type III epoxy concrete at different temperatures shows that this type of epoxy concrete exhibits stability. Therefore, it can be concluded that adding solid asphalt particles can improve the thermal sensitivity of epoxy concrete, ensuring better stress coordination between repair materials and original pavement materials under different temperatures, optimizing the performance of epoxy concrete, and ensuring the stability and durability of rapid bridge deck repair materials under various climatic conditions.

## 4. Conclusions

This study proposed an epoxy concrete material for rapid repair of bridge deck paving, in the field of bridge deck pavement repair materials technology. Through research on the compressive strength, UV aging resistance, and thermal sensitivity of epoxy concrete, the following conclusions were drawn:

(1) The addition of carbon black and rubber powder can increase the compressive strength of epoxy concrete cubes while lowering the elastic modulus, enhancing the toughness of bridge deck paving repair materials. Specifically, the compressive strength of epoxy concrete cubes after 2 h increased by 20.0%, while the elastic modulus decreased by 25.0% with the addition of carbon black and rubber powder.
(2) Adding carbon black can enhance the UV aging resistance of epoxy concrete, thereby potentially increasing the service life of bridge deck paving repair materials. Under UV aging conditions, the addition of carbon black and rubber powder in epoxy concrete cubes increased the compressive strength by 28.6%. Compared to similar epoxy concrete under standard conditions, the decrease in compressive strength of the cubes under UV aging conditions decreased from 78.8% to 76.9%.
(3) The addition of asphalt particles helps improve the thermal sensitivity of epoxy concrete, ensuring better stress coordination between repair materials and original paving materials under different temperatures. When temperatures range from −10 °C to room temperature, the compressive strength and the elastic modulus of the 3-day cube specimens are similar to those at room temperature. However, when the temperature exceeds 40 °C, the cube compressive strength decreased by 58.8% and the elastic modulus decreased by over 52.3%.
(4) Epoxy concrete materials with the addition of carbon black, rubber powder, and rubber particles can cure within two hours, with a compressive strength exceeding 10 MPa, and reach a 3-day cube compressive strength of over 30 MPa. The strength is stable, meeting the early strength requirements for rapid repair materials of bridge deck paving.

## Figures and Tables

**Table 1:** Technical index of epoxy resin.

Name of a Shop	Pigment	Epoxy Value (mol/100 g)	Hydrolysis of Chlorine (%)	Volatile (150 °C/40 min/%)	Viscosity (25 °C/mPa·s)
E51	transparency liquid	0.48–0.54	0.20	0.50	11,000~14,000

**Table 2:** C12–14 technical index of fat glycidyl ether.

Name of a Shop	Pigment	Proportion	Formula Weight	Primary Distillation Point	Flash Point	Toxic Odor
LS-AGE	Colorless transparent liquid	0.89	242~270	>200 °C	>90 °C	low

**Table 3:** Material ratio.

Composition of Aggregate	Specifications	Proportion
Corundum	2–4 eyes	10–30
	8–10 eyes	10–30
	20–40 eyes	10–30
Rubber carbon black	More than 100 eyes	0–2
Rubber powder	More than 100 eyes	0.5–6
Asphalt particles	Of the 80–100 orders	5–15

**Table 4:** Main test equipment and indicators.

Device Name	Model	Technical Indicators and Characteristics
Cement mixer	JJ-5	Rotation (low speed 140 ± 5 r/min, high speed 285 ± 10 r/min). Revolution (low speed 62 ± 5 r/min, high speed 125 ± 10 r/min). Power (0.55/0.37 kw). Slow stirring 30 s, sand 30 s, fast stirring 30 s total 90 s stop 180 s after fast speed, mixing blade and mixing blade shaft connecting thread M18 × 1.5.
Irradiation-based aging test box	FZX-880	Temperature range 80 ± 8 °C. Main peak wavelength 365 nm. Turntable size 500 nm. Power supply 220 V. Power 2 kW*ϕ*.
electrothermal constant-temperature dry box	101A-3	Operating temperature is 10–300 °C. The volume is 50 × 60 × 75 cm^3^. Voltage 220 V. Power 4 kW.
Microcomputer control electrohydraulic servo universal testing machine	WAW-1000	The maximum test force is 1000 kN. The relative error of test force value is within 0.5. The force resolution is 50,000 yards. The deformation measurement range is 2100 FS. The relative error of deformation value is within 0.5. The deformation resolution is 1~100% FS. The power is 380 V, 2 kW.

**Table 5:** Test conditions of epoxy concrete.

Epoxy Concrete Types	Material Ratio							Sample Size (mm)	Number	Curing Condition	Testing Conditions
	Epoxy Resin (%)	Thinner (%)	Hardener (%)	Corundum (%)	Druss (%)	Rubber Powder (%)	Asphalt Particles (%)				
Type I	12	1	12	75	0	0	0	100 × 100 × 100	15	Temperature of 20 ± 2 °C, and a relative humidity of 95%	Temperature of 20 ± 5 °C, relative humidity of not less than 50%
								100 × 100 × 300	15		
Type II	12	1	12	70	1	4	0	100 × 100 × 100	15		
								100 × 100 × 300	15		
Type III	12	1	12	67	1	4	3	100 × 100 × 100	18		
								100 × 100 × 300	18		

**Table 6:** Compressive strength of cubes at different ages.

Composition of Specimen Materials	Age	The Specimen Number	Failing Load (kN)	Compressive Strength of the Cube (MPa)	Average Compressive Strength (MPa)	Standard Deviation (MPa)
Composition of Type I epoxy concrete material	2 h	001	86.4	9	10	1.00
		002	105.6	11		
		003	95.1	10		
	4 h	004	254.8	26	23	2.65
		005	197.6	21		
		006	211.3	22		
	1 d	007	310.5	33	29	3.61
		008	268.9	28		
		009	244.6	26		
	3 d	010	297.7	31	33	2.65
		011	313.6	32		
		012	345.7	36		
Composition of Type II epoxy concrete material	2 h	013	134.5	14	12	2.65
		014	98.0	10		
		015	114.1	12		
	4 h	016	268.9	28	29	2.65
		017	259.3	27		
		018	301.1	32		
	1 d	019	336.1	35	32	2.65
		020	288.1	30		
		021	303.8	31		
	3 d	022	403.4	42	39	2.65
		023	355.3	37		
		024	357.5	38		

**Table 7:** Stressed elastic modulus at different ages.

Composition of Specimen Materials	Age	The Specimen Number	*F*_*a*_ (N)	*F*_0_ (N)	$\varepsilon_{a}$ (mm)	$\varepsilon_{0}$ (mm)	*E*_*c*_ (MPa)	*E*_*c*_ Average Value (MPa)	Standard Deviation (MPa)
Composition of Type I epoxy concrete material	2 h	025	24,955	4735	3.254	0.694	125.11	125.43	0.28
		026	25,377	4802	3.273	0.713	125.53		
		027	25,515	4705	3.312	0.672	125.65		
	4 h	028	65,722	4802	2.253	0.944	726.87	727.43	0.64
		029	65,874	4802	2.243	0.933	728.13		
		030	64,976	4705	2.242	0.921	727.29		
	1 d	031	98,694	4705	1.904	0.567	1120.59	1121.32	1.85
		032	99,101	4802	1.904	0.593	1123.42		
		033	101,032	4802	1.904	0.562	1119.95		
	3 d	034	120,868	4735	1.778	0.353	1290.87	1294.32	3.09
		035	120,741	4802	1.791	0.393	1295.27		
		036	119,902	4705	1.778	0.362	1296.82		
Composition of Type II epoxy concrete material composition	2 h	037	17,538	4802	2.620	0.558	96.47	94.12	2.24
		038	16,880	4705	2.611	0.544	93.89		
		039	16,606	4705	2.629	0.567	92.00		
	4 h	040	73,472	4802	2.348	0.508	582.89	579.42	3.11
		041	71,332	4705	2.353	0.517	578.47		
		042	73,468	4802	2.353	0.494	576.90		
	1 d	043	78,551	4802	1.551	0.457	1052.88	1053.10	1.56
		044	78,008	4802	1.554	0.470	1054.76		
		045	78,433	4735	1.554	0.444	1051.66		
	3 d	046	92,733	4802	1.378	0.222	1188.02	1190.41	3.45
		047	90,646	4705	1.378	0.231	1194.37		
		048	91,424	4802	1.378	0.240	1188.84		

**Table 8:** Compressive strength of cubes under different conditions.

Composition of Specimen Materials	Maintenance Conditions and Age Period	The Specimen Number	Failing Load (kN)	Compressive Strength of the Cube (MPa)	Average Compressive Strength (MPa)	Standard Deviation (MPa)
Composition of Type I epoxy concrete material	Conditions I (for 3 d of curing at room temperature)	010	297.7	31	33	2.65
		011	313.6	32		
		012	345.7	36		
	Condition II (with 1 d curing at room temperature, PV42.2 °C and SV 45.0 °C for two days)	055	76.8	8	7	1.00
		056	66.5	7		
		057	57.6	6		
Composition of Type II epoxy concrete material	Conditions I (for 3 d of curing at room temperature)	046	403.4	42	39	2.65
		047	355.3	37		
		048	357.5	38		
	Condition II (with 1 d curing at room temperature, PV42.2 °C and SV 45.0 °C for two days)	058	107.8	11	9	2.65
		059	96.0	10		
		060	57.6	6		

**Table 9:** The elastic modulus of static force under different conditions.

Composition of Specimen Materials	Maintenance Conditions and Age Period	Specimen Number	*F*_*a*_ (N)	*F*_0_ (N)	$\varepsilon_{a}$ (mm)	$\varepsilon_{0}$ (mm)	*E*_*c*_ (MPa)	*E*_*c*_ Average Value (MPa)	Standard Deviation (MPa)
Composition of Type I epoxy concrete material	Room temperature, 3 d	034	120,868	4735	1.778	0.353	1290.87	1294.32	3.09
		035	120,741	4802	1.791	0.393	1295.27		
		036	119,902	4705	1.778	0.362	1296.82		
	Room temperature for 1 d, PV42.2 °C, and SV 45.0 °C	049	21,286	4802	1.328	0.612	359.57	357.23	2.22
		050	20,134	4705	1.315	0.626	356.96		
		051	21,902	4802	1.346	0.594	355.16		
Composition of Type II epoxy concrete material	Room temperature, 3 d	046	92,733	4802	1.378	0.222	1188.02	1190.41	3.45
		047	90,646	4705	1.378	0.231	1194.37		
		048	91,424	4802	1.378	0.240	1188.84		
	Room temperature for 1 d, PV42.2 °C, and SV 45.0 °C	052	35,889	4735	2.176	0.753	346.78	348.16	1.60
		053	36,997	4802	2.171	0.734	349.92		
		054	35,358	4705	2.185	0.780	347.78		

**Table 10:** Curing conditions and curing age.

Abbreviations	Specific Reference to Abbreviations
C I	Cured for 3 days under conditions of 20 ± 2 °C and 95% relative humidity
C II	Cured for 1 day under conditions of 20 ± 2 °C and 95% relative humidity, then cured for two days at PV 42.2 °C and SV 45.0 °C
T I	Type I epoxy concrete
T II	Type II epoxy concrete

**Table 11:** Compressive strength of Type III epoxy concrete cube at different temperature.

Composition of Specimen Materials	Maintenance Conditions and Age Period	Temperature (°C)	Specimen Number	Failing Load (kN)	Compressive Strength of the Cube (MPa)	Compressive Strength (MPa)	Standard Deviation (MPa)
Composition of Type III epoxy concrete material	At room temperature, 3 d	−10	061	355,348	37	38	1.00
			062	372,438	38		
			063	374,556	39		
		0	064	268,912	28	28	1.00
			065	256,662	27		
			066	278,516	29		
		10	067	240,100	25	25	1.00
			068	230,496	24		
			069	244,634	26		
		20	070	352,836	36	34	2.00
			071	307,328	32		
			072	326,536	34		
		40	073	147,015	15	14	1.00
			074	124,852	13		
			075	134,456	14		
		60	076	123,578	13	11	2.00
			077	86,436	9		
			078	105,644	11		

**Table 12:** The static compression elastic modulus of Type III of epoxy concrete at different temperatures.

Composition of Specimen Materials	Maintenance Conditions and Age Period	Temperature (°C)	The Specimen Number	*E_c_* (MPa)	*E_C_* Average Value (MPa)	Standard Deviation (MPa)
Composition of Type III epoxy concrete material	At room temperature, 3 d	−10	079	1012.56	1014.16	5.02
			080	1019.78		
			081	1010.14		
		0	082	609.58	614.50	4.29
			083	617.43		
			084	616.49		
		10	085	522.66	524.44	5.09
			086	520.48		
			087	530.18		
		20	088	826.79	828.96	1.90
			089	829.78		
			090	830.31		
		40	091	392.36	395.29	2.64
			092	397.48		
			093	396.03		
		60	094	325.26	327.63	2.16
			095	329.49		
			096	328.14		

## Data Availability

The original contributions presented in the study are included in the article, further inquiries can be directed to the corresponding authors.
